# Supplementary material for: Heat adaptation of phage T7 under an extended genetic code
Source: Virus Evol. 2021 Dec 1;7(2):veab100. doi: 10.1093/ve/veab100 (PMC8923235; doi:10.1093/ve/veab100)
Supplement: veab100_Supp [file veab100_supp.zip › Sequencing_Analysis.zip!/Sequencing_Analysis/S1/evidence/JC_SIDE_2_62.html]

BRESEQ :: Evidence


| Predicted mutation | | | | | | |
| --- | --- | --- | --- | --- | --- | --- |
| evidence | seq id | position | mutation | annotation | gene | description |
| JC | NC\_001604.1 | 27,769 | (ATATCTGGCGCT)_1→2_ | intergenic (–/–) | – / – | –/– |

| New junction evidence | | | | | | | | | | | |
| --- | --- | --- | --- | --- | --- | --- | --- | --- | --- | --- | --- |
|  | | seq id | position | reads (cov) | reads (cov) | score | skew | freq | annotation | gene | product |
| \* | ? | NC\_001604.1 | = 27753 | 15 (0.000) | 3703 (0.920) +CGCTATATCTGG | 393/544 | 0.0 | 99.6% | intergenic (–/–) | *–/–* | –/– |
|  | ? | NC\_001604.1 | 27754 = | 18 (0.000) |  |  |  |  | intergenic (–/–) | *–/–* | –/– |

Only 100 of 4154 total aligned reads displayed.  

`CTAGCTATCGGTGGTAACTGCGGGGACCAGTGCTGGTTCGTTACGAGCGACCAAGTGTGGCGACTTAGTGGAAAGGCTAAGCGAAAGTTCCGTAAGTTAATCATGGAGTATCGCGATAAGATGCTTGAGAAGTATGATACTCTTTGGAATTACGTATGGGTAGGCAATACGTCCCACATTCGTTTCCTCAAGACTATCGGTGCGGTATTCCATGAAGAGTACACACGAGATGGTCAATTTCAGTTATTTACAATCACGAAAGGAGGATAACCATATGTGTTGGGCAGCCGCAATACCTATCGCTATATCTGGCGCTCAGGCTATCAGTGGTCAGAACGCTCAGGCCAAAATGATTGCCGCTCAGACCGCTGCTGGTCGTCGTCAAGCTATGGAAATCATGAGGCAGACGAACATCCAGAATGCTGACCTATCGTTGCAAGCTCGAAGTAAACTTGAGGAAGCGTCCGCCGAGTTGACCTCACAGAACATGCAGAAGGTCCAAGCTATTGGGTCTATCCGAGCGGCTATCGGAGAGAGTATGCTTGAAGGTTCCTCAATGGACCGCATTAAGCGAGTCACAGAAGGACAGTTCATTCGGGAA  >  NC_001604.1/27454‑28054`  

                                                                                                                                                                                                                                                                                                            `|`                                                                                                                                                                                                                                                                                                              
`ggtaactgcggggaccagtgctggttcgttacgagcgaccaagtgtgccgacttagtggaaaggctaagcgaaagttccgtaagttaatcatggagtatcgcgataagatgcttgagaagtatgatactctttggaattacgtatgggtaggcaatacgtcccacattcgtttcctcaagactatcggtgcggtattccatgaagagtacacacgagatggtcaatttcagttatttacaatcacgaaaggaggataaccatatgtgttgggcagccgcaatacctatcgctatatctggc                                                                                                                                                                                                                                                                                                              <  1:198658‑M2/1‑1 (MQ=255)`  
       `gcggggaccagtactggttcgttacgagcgaccaagtgtggcgacttagtggaaaggctaagcgaaagttccgtaagttaatcatggagtatcgcgataagatgcttgagaagtatgatactctttggaattacgtatgggtaggcaatacgtcccacattcgtttcctcaagactatcggtgcggtattccatgacgagtacacacgagatggtcaatttcagttatttacaatcacgaaagtaggataaccatatgtgttgggcagccgcaatacctatcgctatatctggcgctatat                                                                                                                                                                                                                                                                                                       >  1:418405‑M2/294‑301 (MQ=255)`  
              `aaggtgctggttcgttacgggcgaccaagtgtggcgaattagtggaaaggctaagcgaaagttccgtaagttaatcatggagtatcgcgataagatgcttgagaagtatgagactctttggaattacgtatgggtaggcaatacgtcccacattcgtttcctcaagactatcggtgcggtattccatgaagagtacacacgagatggtcaatttcagttatttacaatcacgaaaggaggataaccatatgtgttgggcagccgcaatacctatcgctatatctggcgctatatctggcgc                                                                                                                                                                                                                                                                                                <  1:647768‑M2/15‑1 (MQ=255)`  
                 `gtgctggttcgttacgagcgaccaagtgtggcgacttagtggaaaggctaagcgaaagttccgtaagttaatcatggagtatcgcgataagatgcttgagaagtatgatactctttggaattacgtatgggtaggcaatacgtcccacattcgtttcctcaagactatcggtgcggtattccatgaagagtacacacgagatggtcaatttcagttatttacaatcacgaaaggaggataaccatatgtgttgggcagccgcaatacctatcgctatatctggCGCTATATCTGGCGCTCa                                                                                                                                                                                                                                                                                             >  1:160407‑M2/284‑301 (MQ=255)`  
                    `agggttcgttacgagcgaccaagtgtggcgacttagtggaaaggctaagcgaaagttccgtaagttaatcatggagtatcgcgataagatgcttgagaagtatgatactctttggaattacgtatgggtaggcaatacgtcccacattcgtttcctcaagactatcggtgaggtattccatgaagagtacacacgagatggtcaatttcagttatttacaatcacgaaaggaggataaccatatgtgttgggcagccgcaatacctatcgctatatctggCGCTATATCTGGCGCTCAGGc                                                                                                                                                                                                                                                                                          <  1:529983‑M2/21‑1 (MQ=255)`  
                          `gtgtataagagacagagtgtggcgacttagtggaaaggctaagcgaaagttccgtaagttaatcatggagtatcgcgataagatgcttgagaagtatgatactctttggaattacgtatgggtaggcaatacgtcccacattcgtttcctcaagactatcggtgcggtattccatgaagagtacacacgagatggtcaatttcagttatttacaatcacgaaaggaggataaccatatgtgttgggcagccgcaatacctatcgctatatctggCGCTATATCTGGCGCTCAGGCTATCAg                                                                                                                                                                                                                                                                                    <  1:275505‑M2/27‑1 (MQ=255)`  
                          `cgttacgagcgaccaagtgtggcgacttagtggaaaggctaagcgaaagttccgtacgttaatcatgtagtatcgcgataagatgcttgagaagtatgatactctttggaattacgtatgggtaggcaatacgtcacacattcgtttactcaagactatcggtgcggtattccatgaagcgtacacacgcgatggtcaatttcagttatttacaatcacgaaaggagaataaccatatgtcttgggtatccgccattactatcgctatatctggCCTTCTATCTGGCGCTCCGTCTatccg                                                                                                                                                                                                                                                                                    >  1:356865‑M2/275‑299 (MQ=255)`  
                          `cgttacgagcgaccaagtgtggcgactgagtggaaaggctaagcgaaagttccgtaagttaatcatggagtatcgcgataagatgcttgagaagtatgatactctttggaattacgtatgggtaggcaatacgtcccacattcgtttcctcaagactatcggtgcggtattccatgaagagtacacacgagatggtcaatttcagttatttacaatcacgaaaggaggataaccatatgtgttgggcagccgcaatacctatcgctatatctggCGCTATATCTGGCGCTCAGGCTATCAg                                                                                                                                                                                                                                                                                    <  1:330830‑M2/27‑1 (MQ=255)`  
                                 `gacagccaagtgtggcgacttagtggaaaggctaagcgaaagttccgtaagttaatcatggagtatcgcgataagatgcttgagaagtatgatactctttggaattacgtatgggtaggcaatacgtcccacattcgtttcctcaagactatcggtgcggtattccatgaagagtacacacgagatggtcaatttcagttatttacaatcacgaaaggaggataaccatatgtgttgggcagccgcaatacctatcgctatatctggCGCTATATCTGGCGCTCAGGCTATCAGTGGTCAg                                                                                                                                                                                                                                                                             <  1:233667‑M2/34‑1 (MQ=255)`  
                                  `gcgaccaagtgttgcgacttagtggaaaggctaagcgaaagttccgtaagttaatcatggagtatcgcgataagatgcttgagaagtatgatactctttggaattacgtatgggtaggcaatacgtcccacattcgtttcctcaagactatcggtgcggtattccatgaagagtacacacgagatggtcaatttcagttatttacaatcacgaaaggaggataaccatatgtgttgggcagccgcaatacctatcgctatatctggCGCTATATCTGGCGCTCAGGATATCAGTGGTCAGa                                                                                                                                                                                                                                                                            <  1:346441‑M2/35‑1 (MQ=255)`  
                                             `tggcgacttagtggaaaggctaagtgaaagttccgtaagttaatcaaggagtatcgcgataagattcttgagaagtatgatactctttggaattacgtatgggtaggcaatacgtcccacattcgtttcctcaagactatcggtgcggtattccatgaagagtacacacgagatggtcaatttcagttatttacaatcacgaaaggaggataaccatatgtgttgggcagccgcaatacctatcgctatatctggCGCTATATCTGGAGCTCAGGCTATCAGTGGTCAGAACGCTCAGGcc                                                                                                                                                                                                                                                                 <  1:315831‑M2/46‑1 (MQ=255)`  
                                             `gggcgacttagtggaaaggctaagcgaaagttccgtaagttaatcatggagtatcgcgataagatgcttgagaagtatgatactctttggaattacgtatgggtaggcaatacgtcccacattcgtttcctcaagaccatcggtgcggtgttccatgaagagtacacacgagatggtcaatttcagttatttacaatcacgaaaggaggataaccatatgtgttgggcagccgcaatacctatcgctatatctggCGCTATATCTGGCGCTCAGGCTATCAGTGGTCAGAACGCTCAGGcc                                                                                                                                                                                                                                                                 <  1:308935‑M2/46‑1 (MQ=255)`  
                                                        `tggaaaggctaagcgaaagttccgtaagttaatcatggagtatcgcgataagatgcttgagaagtatgatactctttggaattacgtatgggtaggcaatacgtcccacattcgtttcctcaagactatcggtgcggtattccatgaagagtacacacgagatggtcaatttcagttatttacaatcacgaaaggaggataaccatatgtgttgggcagccgcaatacctatcgctatatctggCGCTATTTCTGACGCGCAGGCTATCAGTGTTTAGACGGCTCAGCCCACAATGATTGc                                                                                                                                                                                                                                                      >  1:86624‑M2/245‑301 (MQ=255)`  
                                                                 `aaagcgaaagtgccggaagttgatcatggagtatcgcgataagatgcttgagaaggatgatactctttggaattacgtatgggtaggcaatacgtcccacatgcgtttcctcgagacgatcggtgcggtatgacatgaagagtacacacgagatggtcaatctcagttattgacaatcacgaaaggaggataaccatatgtggtggtcagccgcaatacctatcgctatatctggCGCGATATCTGGCGCTCAGACTAGCAGTGGTCAGAACGCTCAGGTCAAAATGATTGCCGCTCAGAc                                                                                                                                                                                                                                             <  1:238352‑M2/66‑1 (MQ=255)`  
                                                                                                 `atcgcgataacatgcttgagaagtatgatactctttggaattacgtatgggtaggcaatacgtcccacattcgtctcctcaagaccatcggtgcggtattccatgaagagtacacacgagatggtcaatttaagttatttacaatcacgaaaggaggataaccatatgtgttgggcagccgcaatacctatcgctatatctggCGCTATATCTGGCGCTAAGGCTAACAGTGGCCAGAACGCTCAGTCCAAAATGATTACCGCTCAGACCGCTGCTGGTCGTCGTCAAGCTTTGGATAtcc                                                                                                                                                                                                             >  1:390175‑M2/204‑300 (MQ=255)`  
                                                                                                               `cttgagaagtatgatactctttggaattacgtatgggtaggcaatacgtcccacattcgtttcctcaagactatcggtgcggtattccatgaagagtacacacgagatggtcaatttcagttatttacaatcacgaaaggatgataaccatatgtgttgggcagccgcaatacctatcgctatatctggCGCTATATCTGGCGCTCAGGCTATCAGTGGTCAGAACGCTCAGGCCAAAATGATTGCCGCTCAGACCGCTGCTGGTCGTCGTCAAGCTCTGGAAATCATGAGGCAGACGAAc                                                                                                                                                                                               >  1:485332‑M2/190‑301 (MQ=255)`  
                                                                                                                       `gtatgatactctttggaattacgtatgggtaggcaatacgtcccacattcgtttcctcaagactatcggtgcggtattccatgaagagtacacacgagatggtcaatttcagttatttacaatcacgaaaggaggataaccatatgtgttgggcagccgcaatacctatcgctatatctggCGCTATATCTGGCGCTCAGGCTATCAGTGGTCAGAACGCTCAGGCCAAAATGATTGCCGCTCAGACCGCTGCTGGTCGTCGTCAAGCTATGGAAATCATGAGGCAGACGAACATCCAGaa                                                                                                                                                                                       >  1:612801‑M2/182‑301 (MQ=255)`  
                                                                                                                       `gtatgatactctttggaattacgtatgggtaggcaatacgtcccacattcgtttcctcaagactatcggtgcggtattccatgaagagtacacacgagatggtcaatttcagttatttacaatcacgaaaggaggataaccatatgtgttgggcagccgcaatacctatcgctatatctggCGCTATATCTGGCGCTCAGGCTATCAGTGGTCAGAACGCTCAGGCCAAAATGATTGCCGCTCAGACCGCTGCTGGTCGCCGTCAACCtatggacatcatgaggccgcccacctgtccctt                                                                                                                                                                                       >  1:209783‑M2/182‑285 (MQ=255)`  
                                                                                                                         `atgatactcttttgaattacgtatgggtaggcaatacgtcccacattcgtttcctcaagactatcggtgcggtattccatgaagagtacacacgggatggtcaatttcagttatttacaatcacgaaaggaggataaccatatgtgttgggcagccgcaatacctatcgctatatctggCGCTATATCTGGCGCTCAGGCTATCAGTGGTCAGAACGCTCAGGCCAAAATGATTGCCGCTCAGACCGCTGCTGGTCGTCGTCAAGCTATGGAAATCATGAGGCAGACGAACATCCAGAATg                                                                                                                                                                                     <  1:14854‑M2/122‑1 (MQ=255)`  
                                                                                                                         `atgatactctttggaattacgtatgggtaggcaatacgtcccacattcgtttcctcaagactatcgttgcggtattccatgaagagtacacacgagatgttcaatttcagttatttacaatcacgaaaggaggataaccctatgtgttgggcagccgcaatacctatcgctatatctggCGCTATATCTGGCGCTCAGGCTATCAGTGGTCAGAACGCTCAGGCCAAAATGATTGCCGCTCAGACCGCTGCTGGTCGTCGTCAAGCTATGGAAACCATGAGGCAGACGAACACCcagattt                                                                                                                                                                                     >  1:81415‑M2/180‑298 (MQ=255)`  
                                                                                                                         `atgatactctttggaattacgtatgggtaggcaatacgtcccacattcgtttcctcaagactatcggttcggtattccatgaagagtacacacgagatggtcaatttcagttatttacaatcacgaaaggaggataaccatatgtgttgggcagccgcaatacctatcgctatatctggCGCTATATCTGGCGCTCAGGCTATCAGTGGTCAGAACGCTCAGGCCAAAAAGATTGCCGCTCAGACCGCTGCTGGCCGTCGTCAAGCTAGGGAAATCATGAGGCAGACGAACATACAGAATg                                                                                                                                                                                     >  1:39563‑M2/180‑301 (MQ=255)`  
                                                                                                                         `atgatactctttggaattacgtatgggtaggcaatacgtcccacattcgtttcctcaagactatcggtgcggtattacatgaagagtacacacgagatggtcaatttcagttatttacaatcacgaaaggaggataaccatatgggttgggcagacgcaatacctatcgctatatctggCGCTATATCTGGCGCTAAGGCTATCAGTGGTCAGAACGCTCAGGGCAAAAAGATTGCCGCTCAGACCGCTGCTGGACGTCGTCAAGCTATGGAAATCATGAGGAAGAAGAACATCCAGAATg                                                                                                                                                                                     >  1:519660‑M2/180‑301 (MQ=255)`  
                                                                                                                          `tataagagacaggaattacgtatgggtaggcaatacgtcccacattcgtttcctcaagactatcggtgcggtattccatgaagagtacacacgagatggtcaatttcagttatttacaatcacgaaaggaggataaccatatgtgttgggcagccgcaatacctatcgctatatctggCGCTATATCTGGCGCTCAGGCTATCAGTGGTCAGAACGCTCAGGCCAAAATGATTGCCGCTCAGACCGCTGCTGGTCGTCGTCAAGCTATGGAAATCATGAGGCAGACGAACATCCAGAATGc                                                                                                                                                                                    <  1:605452‑M2/123‑1 (MQ=255)`  
                                                                                                                              `gagacagggaattacgtatgggtaggcaatacgtcccacattcgtttcctcaagactatcggtgcggtattccatgaagagtacacacgagatggtcaatttcagttatttacaatcacgaaaggaggataaccatatgtgttgggcagccgcaatacctatcgctatatctggCGCTATATCTGGCGCTCAGGCTATCAGTGGTCAGAACGCTCAGGCCAAAATGATTGCCGCTCAGACCGCTGCTGGTCGTCGTCAAGCTATGGAAATCATTAGGCAGACGAACATCCAGAATGCTGAc                                                                                                                                                                                <  1:567460‑M2/127‑1 (MQ=255)`  
                                                                                                                                 `ctttggaattacgtatgggtaggcaatacgtcccacattcgtttcctcaagactatcggtgcggtattccatgaagagtacacacgagatggtcaatttcagttatttacaatcacgaaaggaggataaccatatgtgttgggcagccgcaatacctatcgctatatctggCGCTATATCTGGCGCTCAGGCTATCAGTGGTCAGAACGCTCAGGCCAAAATGATTGCCGCTCAGACCGCTGCTGGTCGTCGTCAAGCTATGGAAATCATGAGGCAGACgaacatccagaactgtctctta                                                                                                                                                                             >  1:193470‑M2/172‑291 (MQ=255)`  
                                                                                                                                 `ctttggaattacgtatgggtaggcaatacgtcccacattcgtttcctcaagactatcggtgcggtattccatgaagagtacacacgagatggtcaatttcagttatttacaatcacgaaaggaggataaccatatgtgttgggcagccgcaatacctatcgctatatctggCGCTATATCTGGCGCTCAGGCTATCAGTGGTCAGAACGCTCAGGCCAAAATGATTGCCGCTCAGACCGCTGCTGGTCGTCGTCAAGCTATGGAAATCATGAGGCAGACGAACCTCCAGAATGCTGACCTa                                                                                                                                                                             >  1:129048‑M2/172‑301 (MQ=255)`  
                                                                                                                                      `gaattacgtatgggtaggcaatacgtcccacattcgtttcctcaagactatcggtgcggtattccatgaagagtacacacgagatggtcaatttcagttatttacaatcacgaaaggaggataaccatatgtgttgggcagccgcaatacctatcgctatatctggCGCTATATCTGGCGCTCAGGCTATCAGTGGTCAGAACGCTCAGGCCAAAATGATTGCCGCTCAGACCGCTGCTGGTCGTCGTCAAGCTATGGAAATCATGAGGCAGACGAACATCCAGAATGCTGACCTATCGtt                                                                                                                                                                        >  1:514646‑M2/167‑301 (MQ=255)`  
                                                                                                                                      `gaattacgtatgggtaggcaatacgtcccacattcgtttcctcaagactatcggtgcggtattccatgaagagtacacacgagatggtcaatttcagttatttacaatcacgaaaggaggataaccatatgtgttgggcagccgcaatacctatcgctatatctggCGCTATATCTGGCGCTCAGGCTATCAGTGGTCAGAACGCTCAGGCCAAAATGATTGCCGCTCAGACCGCTGCTGGTCGTCGTCAAGCTATGGAAATCATGAGGCAGACGAACATCCAGAATGCTGACCTATCGtt                                                                                                                                                                        >  1:93538‑M2/167‑301 (MQ=255)`  
                                                                                                                                           `acgtatgggtaggtaatacgtcccacattcgtttcctcaagactatcggtgcggtattccatgaagagtacacacgagatggtcaatttcagttatttacaatcacgaaaggaggataaccatatgtgttgggcagccgcaatacctatcgctatatctggCGCTATATTTGGCGCTCAGGCTATCAGTGGTCAGAACGCTCAGGCCAAAATGATTGCCGCTCAGACCGCTGCTGGTCGTCGTCAAGCTATGGAAATCATGAGGCAGACGAACATCCAGAATGCTGACCTATCGTTGCAAg                                                                                                                                                                   >  1:74696‑M2/162‑301 (MQ=255)`  
                                                                                                                                           `acgtatgggtaggcaatacgtcccacattcgtttcctcaagactatcggtgcggtattccatgaagagtacacacgagatggtcaatttcagttatttacaatcacgaaaggaggataaccatatgtgttgggcagccgcaatacctatcgctatatctggCGCTATATCTGGCGCTCAGGCTATCAGTGGTCAGAACGCTCAGGCCAAAATGATTGCCGCTCAGACCGCTGCTGGTCGTCGTCAAGCTATGGAAATCATGAGGCAGACGAACATCCAGAATGCTGACCTATCGTTGCAAg                                                                                                                                                                   >  1:25636‑M2/162‑301 (MQ=255)`  
                                                                                                                                                `tgggtaggcaatacgtcccacattcgtttcctcaagactatcggtgcggtattccatgaagagtacacacgagatggttaatttcagttatttacaatcacgaaaggaggataaccatatgtgttgggcagccgcaatacctatcgctatatatggCGCTATATCTGGCTCTCAGGCTACCAGTGGTCAGAACGCTCCGGCCAAAAGGATTGCCGCTCAGACCGCTGCTGGTCGTCGTCAAGCTATGGAAATCATGAGGCAGACGAACATACAGAATGCTGACCTATCGTTGCAACCACGa                                                                                                                                                              >  1:537075‑M2/157‑301 (MQ=255)`  
                                                                                                                                                      `ggcaatacgtcccacattcgtttcctcaagactatcggtgcggtattccatgaagagtacacacgagatggtcaatttcagttatttacaatcacgaaaggaggataaccatatgtgttgggcagccgcaatacctatcgctatatctggCGCTATATCTGGCGCTCAGGCTATCAGTGGTCAGAACGCTCAGGCCAACATGATTGCCGCTCAGACCGCTGCTGGTCGTCGTCAAGCTATGGAAATCACGAGGCAGACGAACATCCAGAATGCTGACCTATCGTTGCAAGCTCGAAGTtaa                                                                                                                                                        >  1:600431‑M2/151‑301 (MQ=255)`  
                                                                                                                                                        `gtgggctcggagatgtgtataagagacagctatcggtgcggtattccatgaagagtacacacgagatggtcaatttcagttatttacaatcacgaaaggaggataaccatatgtgttgggcagccgcaatacctatcgctatatctggCGCTATATCTGGCGCTCAGGCTATCAGTGGTCAGAACGCTCAGGCCAAAATGATTGCCGCTCAGACCGCTGCTGGTCGTCGTCAAGCTATGGAAATCATGAGGCAGACGAACATCCAGAATGCTGACCTATCGTTGCAAGCTCGAAGTAAACt                                                                                                                                                      <  1:594102‑M2/153‑1 (MQ=255)`  
                                                                                                                                                          `atacttcccacattcttttccgcaagactatgtgtgcggtattccatgaagagtacacacgagatggtcaatttcagttattgacaatcacgaaaggaggataaccatatgtgttgggcagccgcaatacctatcgctatatctggCGCTATATCTGGCGCTCAGGCTATCAGTGGTCAGAACGCTCAGGCCAAAATGATTGCCGCTCAGACCGCTGCTGGTCGTCGTCAAGCTATGGAAATCATGAGGCAGACGAACATCCAGAATGCTGACCTATCGTTGCAAGCTCGAAGTAAACTTg                                                                                                                                                    <  1:653559‑M2/155‑1 (MQ=255)`  
                                                                                                                                                              `tgacagcactcttttcctaaaggctaccggtgcggtattccattaagagtagacacgagatgatcaatttctgttatttacaatcacgaaaggatgatcaccatatgtgtggggcagccgcaatacctatctcaatatcgttCGCTAGATCTGGCGCTCAGGCTATCAGTGGTCAGAACGCGCAATCCAAAATGATAGCCGCTCAGACCTCTGCTGGTCGGCGTCAAGCTATGGAAATCATAATGCAGACGAACATCCAGAATGCTGACCTATCGTTGCAAGCTCGAAGTAAACTTGAGGa                                                                                                                                                <  1:351464‑M2/159‑1 (MQ=255)`  
                                                                                                                                                              `gtcccacattcgtttcctcaagactatcggtgcggtattccatgaagagtacacacgagatggtcaatttcagttatttacaatcacgaaaggaggataaccatatgtgttgggcagccgcaatagctatcgctatatctggCGCTATATCTGGCGCTCAGGCTATTAGTGGTCAGAACGCTCAGGCCAAAATGAGTGCCGCTCAGACCGCTGCTGGTCGTCGTCAAGCTATGTAAATCATGAGGCAGACGAACATCCAGAATGCTGACCTATCGTTGCAAGCTCGAAGTAAACTTGAGGa                                                                                                                                                <  1:49024‑M2/159‑1 (MQ=255)`  
                                                                                                                                                              `ctcccaccttcgtttccgccagactatcggtgcggtattccatgaagagtacacacgagatggtcaatttcagttatttacaatcacgaaaggaggataaccatatgtgttgggcagccgcaatacctatcgctatatctggCGCTATATCTGGCGCTCAGGCTATCAGTGGTCAGAACGCTCAGGCCAAAATGATTGCCGCTCAGACCGCTGCTGGTCGTCGTCAAGCTATGGAAATCATGAGGCAGACGAACATCCAGAATGCTGACCTATCGTTGCAAGCTCGAAGTAAACTTGAGGa                                                                                                                                                <  1:618086‑M2/159‑1 (MQ=255)`  
                                                                                                                                                               `tcccacattcgtttcatcaagactatcggtgcggtattccatgaagagtacacacgagctgttcaatttccgttatttacaatcacgaaaggaggataaccatatgtgttaggcagccgcaatatctatcgctatatctggCGCTATATCTGTCTCTCAGGCTATCAGTGGTCAGAACGCTCAGGCCAAAATGATTGCCGCTCAGACCGATGCTGGTCGTCGTCAAGCTATGGAACTCATGAGGCAGACGAACATCCAGAATGCTGACCTATCGTTGCAAGCTCGAAGTAAACTTCAGGaa                                                                                                                                               >  1:46751‑M2/142‑301 (MQ=255)`  
                                                                                                                                                                             `cctcaagactatcggtgcggtattccatgaagagtacacacgagatggtcaatttcagttatttacaatcacgaaaggaggataaccatatgtgttgggcagccgcaatacctatcgctatatctggCGCTATATCTGGCGCTCAGGCTATCAGTGGTCAGAACGCTCAGGCCAAAATGATTGCCGCTCAGACCGCTGCTGGTCGTCGTCAAGCTATGGAAATCATGAGGCAGACGAACATCCAGAATGCTGACCTATCGTTGCAAGCTCGAAGTAAACTTTAGGAAGCGTCCGCCGAGtt                                                                                                                                 >  1:167826‑M2/128‑301 (MQ=255)`  
                                                                                                                                                                              `ctcaagactatcggtgcggtattccatgaagagtacacacgagatggtcaatttcagttatttacaatcacgaaaggaggataaccatatgtgttgggcagccgcaatacctatcgctatatctggCGCTATATCTGGCGCTCAGGCTATCAGTGGTCAGAACGCTCAGGCCAAAATGATTGCCGCTCAGACCGCTACTGGTCGTCGTCAAGCTATGGAAATCATGAGGCAGACGAACATCCAGAATGCTGACCAATCGTTGCAAGCTCGAAGTAAACTTTAGGAAGCGTCTGCCGAGTTg                                                                                                                                >  1:668433‑M2/127‑301 (MQ=255)`  
                                                                                                                                                                                  `gatgtgtataagagacagtccatgaagagtacacacgagatggtcgatttcagttatttacaatcacgaaaggaggataaccatatgtgttgggcagccgcaatacctatcgctatatctggCGCTATATCTGGCGATCAGGCTATCAGTGGTCAGAACGCTCAGGCCAAAATGATTGCCGCTCAGACCGCTGCTGGTCGTCGTCAAGCTATGGAAATCATGAGGCAGACGAACATCCAGAATGCTGACCTATCGTTGCAAGCTCGAAGTAAACTTGAGGAAGCGTCCGCCGAGTTGaccc                                                                                                                            <  1:270663‑M2/179‑2 (MQ=255)`  
                                                                                                                                                                                   `gactatcggtgcggtattccatgaagagtacacacgagatggtcaatttcagttatttacaatcacgaaaggaggataaccatatgtgttgggcagccgcaatacctatcgctatatctggCGCTATATCTGGCGCTCAGGCTATCAGTGGTCAGAACGCTCAGGCCAAAATGATTGCCGCTCAGACCGCTGCTGGTCGTCGTCAAGCTATGGAAATCATAAGGCAGACGAACATCCAGCATGCTGACCTATCGTTGCAAGCTCGAAGTAAACTTGAGGAAGCGTCCGCCGAGTTtacccc                                                                                                                           >  1:447983‑M2/122‑299 (MQ=255)`  
                                                                                                                                                                                     `ctatcggtgcggtattccatgaagagtacacacgagatggtcaatttcagttatttacaatcacgaaaggaggataaccatatgtgttgggcagccgcaatacctatcgctatatctggCGCTATATCTGGCGCTCAGGCTATCAGTGGTCAGAACGCTCAGGCCAAAATGATTGCCGCTCAGACCGCTGCTGGTCGTCGTCAAGCTATGGAAATCATGAGGCACACGAACATCCAGAATGCTGACCTATCGTTGCAAGCTCGAAGTAAAATTGAGGAAACGTCCGCCGAGTTGACCCcac                                                                                                                         >  1:400647‑M2/120‑301 (MQ=255)`  
                                                                                                                                                                                     `acatccgtgcggtatgccatgaagagtacacccgagatagtcaatttcagttatttacaatcacgaaaggaggataaccatatgtgttggccagccgcaatacctatccctatatctggCGCTATATCTGGCGCTCATGCTATCAGTGGTCAGAACGCTCAGGCCAAAATGATTGCCGCTCAGACCGCTGCTGGTCGTCGTCAAGCTATGGAAATCATGAGGCAGTCGAACATCCAGAATGCTGACCTATCGTTGCAAGCTCGAAGTAAACTTGAGGAAGCGTCCGCCGAGTTGACCCcac                                                                                                                         <  1:623999‑M2/182‑1 (MQ=255)`  
                                                                                                                                                                                          `ggtgcggtattccatgaagagtacacacgagatggtcaatttcagttatttacaatcacgaaaggaggataaccatatgtgttgggcagccgcaatacctatcgctatatctggCGCTATATCTGGCGCTCAGGCTATCAGTGGTCAGAACGCTCAGGCCAAAATGATTGCCGCTCAGACCGCTGCTGGTCGTCGTCAAGCTATGGAAATCATGAGGCAGACGAACAACCAGAATGCTGACCTATCGTTACAAGCTCGAAGTAAACTTGAGGAAGCGTCCGCCGAGTTGACCCCACAGAAc                                                                                                                    <  1:1430‑M2/187‑1 (MQ=255)`  
                                                                                                                                                                                           `gtgcggtattccatgaagagtacacacgagatggtcaatttcagttatttacaatcacgaaaggaggataaccatatgtgttgggcagccgcaatacctatcgctatatctggCGCTATATCTGGCGCTCAGGCTATCAGTGGTCAGAACGCTCAGGCCAAAATGATTGCCGCTCAGACCGCTGCTGGTCGTCGTCAAGCTATGGAAATCATGAGGCAGACGAACATCCAGAATGCTGACCTATCGTTGCAAGCTCGAAGTAAACTTGAGGAAGCGTCCGCCGAGTTGACCCCACAGAACa                                                                                                                   <  1:120649‑M2/188‑1 (MQ=255)`  
                                                                                                                                                                                           `gtgcggtattccatgaagagtacacacgagatggtcaatttcagttatttacaatcacgaaaggaggataaccatatgtgttgggcagccacaatacctatcgctatatctggCGCTATATCTGGCGCTCAGGCTATCAGTGGTCAGAACGCTCAGGCCAAAATGATTGCCGCTCAGACCGCTGCTGGTCGTCGTCAAGCTATGGAAATCATGAGGAAGACGAACATCCAGAATGCTGACCTATCGTTGCAAGCTCGAAGTAAACTTGAGGAAGCGTCCGCCGAGTTGACCTcacagctgt                                                                                                                   >  1:227745‑M2/114‑297 (MQ=255)`  
                                                                                                                                                                                             `gcggtattccatgaagagtacacacgagatggtcaatttcagttatttacaatcacgaaaggaggataaccatatgtgttgggaagccgcaatacctatcgctatatctggCGCTATATCTGGCGCTCAGGCTATCAGTGGTCAGAACGCTCAGGCCAAAATGATTGCCGCTCAGACCGCTGCTGGTCGTCTTCAAGCTCTGGAAATAATGAGTCAGACGCACAACCAGAATGCTGACCTATCGTTGCAAGCTCGAAGTAAACTTGAAGAAGCGACCGCTGCGTTGAACACCCAGAACATg                                                                                                                 >  1:371506‑M2/112‑301 (MQ=255)`  
                                                                                                                                                                                                 `gtgtataagagacagacacacgagatggtcaatttcagttatttacaatcacgaaaggatgataaccatatgtgttgggcagccgcaatacctatcgctatatctggCGCTATATCTGGCGCTCAGGCTATCAGTGGTCAGAACGCTCAGGCCAAAATGATTGTCGCTCAGACCGCTGCTGGTCGTCGTCAAGCTATGGAAATCATGAGGCAGACGAACATCCAGAATGCTGACCTATCGTTGCAAGCTCGAAGTAAACTTGAGGAAGCGTCCGCCGAGTTGACCCCACAGAACATGCAGa                                                                                                             <  1:297905‑M2/194‑1 (MQ=255)`  
                                                                                                                                                                                                 `cattccatgaagagtacacacgagatggtcaatttcagttatttacaatcacgaaaggaggataaccatatgtgttgggcagccgcaatacctatcgctatatctggCGCTATATCTGGCGCTCAGGCTATCAGTGGTCAGAACGCTCAGGCCAAAATGATTGCCGCTCAGACCGCTGCTGGTCGTCGTCAAGCTATGGAAATCATGAGGCAGACGAACATCCAGAATGCTGACCTATCGTTGCAAGCTCGAAGTAAACTTGAGGAAGCGTCCGCCGAGTTGACCCCACAGAACATGCAGa                                                                                                             <  1:662952‑M2/194‑1 (MQ=255)`  
                                                                                                                                                                                                  `attccatgaagagtacacacgagatggtcaatttcagttatttacaatcacgaaaggaggataaccatatgtgttgggcagccgcaatacctatcgctatatctggCGCTATATCTGGCGCTCAGGCTATCAGTGGTCAGAACGCTCAGGCCAAAATGATTGCCGCTCAGACCGCTGCTGGTCGTCGTCAAGCTATGGAAATCATGAGGCAGACGAACATCCAGAATGCTGACCTATCGTTGCAAGCTCGAAGTAAACTTGAGGAAGCGTCCGCCGAGTTGACCCCACAGaacctgtctct                                                                                                            >  1:525283‑M2/107‑296 (MQ=255)`  
                                                                                                                                                                                                    `tccatgaagagtacacacgagatggtcaatttcagttatttacaatcacgaaaggaggataaccatatgtgttgggcagccgcaatacctatcgctatatctggCGCTATATCTGGCGCTCAGGCTATCAGTGGTCAGAACGCTCAGGTCAAAATGATTGCCGCTCAGACCGCTGCTGGTCGTCGTCAAGCTATGGAAATCATGAGGCAGACGAACATCCAGAATGCTGACCTATCGTTGCAAGCTCGAAGTAAACTTGAGGAAGCGTCCGCCGAGTTGACCCCACAGAACATGCAGAAgg                                                                                                          >  1:221679‑M2/105‑301 (MQ=255)`  
                                                                                                                                                                                                    `tccatgaagagtacacacgagatggtcaatttcagttatttacaatcacgaaaggaggataaccatatgtgttgggcagccgcaatacctatcgctatatctggCGCTATATCTGGCGCTCAGGCTATCAGTGGTCAGAACGCTCAGGCCAAAATGATTGCCGCTCAGACCGCTGCTGGTCGTCGTCAAGCTATGGAAATCATGAGGCAGACGAACATCCAGAATGCTGACCTATCGTTGCAAGCTCGAAGTAAACTTGAGGAAGCGTCCGCCGAGTTGACCCCACAGAACATGCAGAAgg                                                                                                          >  1:507716‑M2/105‑301 (MQ=255)`  
                                                                                                                                                                                                         `gaagagtacacacgagatggtcaatttcagttatttacaatcacgaaaggaggataaccatatgtgttgggcagccgcaatacctatcgctatatctggCGCTATATCTGGCGCTCAGGCTATCAGTGGTCAGAACGCTCAGGCCAAAATGATTGCCGCTCAGACCGCTGCTGGTCGTCGTCAAGCTATGGAAATCATGAGGCAGACGAACATCCAGAATGCTGACCTATCGTTGCAAGCTCGAAGTAAACTTGAGGAAGCGTCCGCCGAGTTGACCCCAACGAACATGCAGCAGGTCCaa                                                                                                     >  1:113288‑M2/100‑301 (MQ=255)`  
                                                                                                                                                                                                            `gagtacacacgagatggtcaatttcagttatttacaatcacgactggaggataaccatatgtgttgggcagccgcaataactatcgccatatctggCGCTATATCTGGCGCTCAGGCTAACAGTGGTAAGAACGCTAAGGCCAAAATGATTGCCGCTCAGACAGCTGCTGGGAGTAGTCAAGCTATGGAATACACGAGGCAGACGATACTCCAGAATGCTGACCTATCGATGAAAGCTCGAAGCAAACTTGAGGAAGCGTGCGCAGCGCTGACCCCACAGAACGTGCAGAAGGTCCAAgca                                                                                                  >  1:181790‑M2/97‑300 (MQ=255)`  
                                                                                                                                                                                                            `gagtacacacgagatggtcaatttcagttatttacaatcacgaaaggaggataaccatatgtgttgggcagccgcaatacctatcgctatatctggCGCTATATCTGGCGCTCAGGCTATCAGTGGTCAGAACGCTCGGGCCAAAATGATTGCCGCTCAGACCGCTGCTGGTCGTCGTCAAGCTATGGAAATCATGAGGCAGACGAACATCCAGAATGCTGACCTATCGTTGCAAGCTCGAAGTAAACTTGAGGAAGCGTCCGCCGAGTTGACCCCACAGAACATGCAGAAGGTCCAAgcc                                                                                                  >  1:285946‑M2/97‑300 (MQ=255)`  
                                                                                                                                                                                                            `gagtacacacgagatggtcaatttcagttatttacaatcacgaaaggaggataaccatatgtgttgggcagccgcaatacctatcgctatatctggCGCTATATCTGGCGCTCAGGCTATCAGTGGTCAGAACGCTCAGGCCAAAATGATTGCCGCTCAGACCGCTGCTGGTCGTCGTCAAGCTATGGAAATCATGAGGCAGCCGAACATCAAGAATGCTGACCTATCGTTGCAAGCTCGAAGCAAACTTGAGGAAGCGTCCGCCGAGTTGACCCCACAGAACATGCAGAAGGTCCAAGCt                                                                                                  >  1:323610‑M2/97‑301 (MQ=255)`  
                                                                                                                                                                                                            `gagtacacacgagatggtcaatttcagtgattgacaatcacgaaaggaggataaccatatgtgtggagcagccgcaatacctatcgctatatctggCGCTATATCTGGCGCTCAGGCTATCAGTGGTCAGAACGCTCAGGCCAAAATGATTGCCGCTCAGTCCGCTGCTGGTCGTTGTCAAGCTATGGAAATCATGAGGCAGACGAACATCCAGAATACTGACCTATCGTTGCAAGCTCGAAGTAAACTTGAGGAAGCGTCCGCCGAGTTGACCCCACAGAACATGCAGAAGGTCCAAGCt                                                                                                  <  1:13568‑M2/205‑1 (MQ=255)`  
                                                                                                                                                                                                            `ataagagacatagatggtcaatttcagttatttacaatcacgaaaggaggataaccatatgtgttgggcagccgcaatacctatcgctatatctggCGCTATATCTGGAGCTCAGGCTATCAGTGGTCAGAACGCTCAGGCCAAAATGATTGCCGCTCAGACCGCTGCTGGTCGTCGTAAAGCTATGGAAATCATGAGGCAGACGAACATCCAGAATGCTGACCGATCGTTGCAAGCTCGAAGTAAACTTGAGGAAGCGTCCGCCGAGTTGACCCCACAGAACATGCAGAAGGTCCAAGCt                                                                                                  <  1:364379‑M2/205‑1 (MQ=255)`  
                                                                                                                                                                                                            `aagtacacacgagatggtcaatttcagttatttacaatcacgaaaggaggataaccatatgtgttgggcagccgcaatacctatcgctatatctagCGCTATATCTGGCGCTCAGGCTATCAGTGGTCAGAACGCTCAGGCCAAAATGATTGCCGCTCAGACCGCTGCTGGTCGTCGTCAAGCTATGGAAATCATGAGGCAGACGAACATCCAGAATGCTGACCTATCGTTGCAAGCTCGAAGTAAACTTGAGGAAGCGTCCGCCGAGTTGAACCCACAGAACATGCAGAAGGTCCAAGCt                                                                                                  <  1:423271‑M2/205‑1 (MQ=255)`  
                                                                                                                                                                                                              `gtacacacgagatggtcaatttcagttatttacaatcacgaaaggaggataaccatatgtgttgggcagccgcaatacctatcgctatatctggCGCTATATCTGGCGCTCAGGCTATCAGTGGTCAGAACGCTCAGGCCAAAATGATTGCCGCTCAGACCGCTGCTGGTCGTCGTCAAGCTATGGAAATCATGAGGCAGACGAACATCCAGAATGCTGACCTATCGTTGCAATCTCGAAGTAAACTTGAGGAAGCGTCCGCCGAGTTGACCCCACAGAACATGCAGAAGGTCCAAGCTAt                                                                                                >  1:428676‑M2/95‑301 (MQ=255)`  
                                                                                                                                                                                                              `gtacacacgagatggtcaatttcagttatttacaatcacgaaaggaggataaccatatgtgttgggcagccgcaatacctatcgctatatctggCGCTATATCTGGCGCTCAGGCTATCAGTGGTCAGAACGCTCAGGCCAAAATGATTGCCGCTCAGACCGCTGCTGGTCGTCGTCAAGCTATGGAAATCATGAGGCAGACGAACATCCAGAATGCTGACCTATCGTTGCAAGCTCGAAGTAAACTTGAGGAAGCGTCCGCCGAGTTGACCCCACAGAACATGCACAAGGTCCAAGCTAt                                                                                                >  1:70282‑M2/95‑301 (MQ=255)`  
                                                                                                                                                                                                                   `cacgagatggtcaatttcagttatttacaatcacgaaaggaggataaccatatgtgttgggcagccgcaatacctatcgctatatctggCGCTATATCTGGCGCTCAGGCTATCAGTGGTCAGAACGCTCAGGCCAAAATGATTGCCGCTCAGACCGCTGCTGGTCGTCGTCAAGCTATGGAAATCATGAGGCAGACGAACATCCAGAATGCTGACCTATCGTTGCAAGCTCGAAGTAAACTTGAGGAAGCGTCCGCCGAGTTGACCCCACAGACCATGCAGAAGGtccaagctctgtctc                                                                                           >  1:440970‑M2/90‑294 (MQ=255)`  
                                                                                                                                                                                                                    `acgagatggtcaatttcagttatttacaatcacgaaaggaggataaccatatgtgttgggcagccgcaatacctatcgctatatctggCGCTATATCTGGCGCTCAGGCTATCAGTGGTCAGAACGCTCAGGCCAAAATGATTGCCGCTCAGACCGCTGCTGGTCGTCGTCAAGCTATGGAAATCATGAGGCAGACGAACATCCAGAATGCTGACCTATCGTTGCAAGCTCGAAGTAAACTTGAGGAAGCGTCCGCCGAGTTGACCCCACAGAACATGCAGAAGGTCCAAGCTATTGGGTc                                                                                          <  1:406983‑M2/213‑1 (MQ=255)`  
                                                                                                                                                                                                                          `tgtgtaatttcagttaattaccatcacgaaaggagcataaccatatgtgttgggcagccgcaataccaatcgctaagtctggCTCTATATCTGGCGCTCAGGTTATCAGTGGTCAGAACGCTCGGGCCAAAATGATTGCCGCTCAGACCGCTGCTGGTCTTCGTCAAGCTATGGAAATCATGAGGCAGACGAACATCCAGTATGCTGACCTATCGTTGCAAGCTCGAAGTAAACTTGAGGAAGCGTCCGCCGAGTTGACCCCACTGAACATGCAGAAGGTCCAAGCTATTGGGTCTATCCg                                                                                    <  1:38405‑M2/219‑1 (MQ=255)`  
                                                                                                                                                                                                                          `tggtcaatttcagttatttacaatcacgaaaggaggataaccatatgtgttgggcagccgcaatacctatcgctatatctggCGCTATATCTGGCGCTCAGGCTATCAGTGGTCAGAACGCTCAGGCCAAAATGATTGCCGCTCAGACCGCTGCTGGACGTCGTCAAGCTATGGAAATCATGAGGCAGACGAACATCCAGAATGCTGACCTATCGTTGCAAGCTCGAAGTAAACTTGAGGAAGCGTCCGCCGAGTTGACCCCACAGAACATGCAGAAGGTCCAAGCTATTGGGTCTATCCg                                                                                    <  1:496‑M2/219‑1 (MQ=255)`  
                                                                                                                                                                                                                           `ggtcaatttcagttatttacaatcacgaaaggtggataaccatatgtgttgggcagccgcaatacctatcgctatatctggCGCTATATCTGGCGCTCAGGCTATCAGTGGTCAGAACGCTCAGGCCAAAATGATTGCCGCTCAGACCGCTGCTGGTCGTCGTCAAGCTATGGAAATCATTAGGCAGACGAACATCCAGAATGCTGACCTATCGTTGCAAGCTCGAAGTAAACTTGAGGAAGCGTCCGCCGAGTTGACCCCACAGACCATGCAGAAGGCACAAGCTAGTGGGTCTATCCGa                                                                                   >  1:454033‑M2/82‑301 (MQ=255)`  
                                                                                                                                                                                                                            `gtcaatttcagttatttacaatcacgaaaggaggataaccatatgtgttgggcagccgcaatacctatcgctatatctggCGCTATATCTGGCGCTCAGGCTATCAGTGGTCAGAACGCTCAGGCCAAAATGATTGCCGCTCAGACCGCTGCTGGTCGTCGTCAAGCTATGGAAATCATGAGGCAGACGAACATCCAGAATGCTGACCTATCGTTGCAAGCTCGAAGTAAACTTGAGGAAGCGTCCGCCGAGTTGACCCCACAGAACATGCAGAAGGTCCAAGCTATTGGGTCTATCCGAg                                                                                  <  1:203346‑M2/221‑1 (MQ=255)`  
                                                                                                                                                                                                                                `atttcagttatttacaatcacgaaaggaggataaccatatgtgttgggcagccgcaatacctatcgctatatctggCGCTATATCTGGCGCTCAGGCTATCAGTGGTCAGAACGCTCAGGCCAAAATGATTGCCGCTCAGACCGCTGCTGGTCGTCGTCAAGCTATGGAAATCATGAGGCAGACGAACATCCAGAATGCTGACCTATCGTTGCAAGCTCGAAGTAAAATTGAGGAAGAGTCCGCCGAGTTGACCCCCCCGACGATGAAGAAGGTACAAGCCTTTGTGTCTTTACGAGCGGc                                                                              >  1:106129‑M2/77‑301 (MQ=255)`  
                                                                                                                                                                                                                                 `tttcagttatttacaatcacgaaaggaggataaccatatgtgttgggcagccgcaatacctatcgctatatctggCGCTATATCTGGCGCTCAGGCTATCAGTGGTCAGAACGCTCAGGCCAAAATGATTGCCGCTCAGACCGCTGCTGGTCGTCGTCAAGCTATGGAAATCATGAGGCAGACGAACATCCAGAATGCTGACCTATCGTTGCAAGCTCGAAGTAAACTTGAGGAAGCGTCCGCCGAGTTGACCCCACAGAACATGCAGAAGGTCCAAGCTATTGGGTCTATCCGAGCGGCt                                                                             >  1:588029‑M2/76‑301 (MQ=255)`  
                                                                                                                                                                                                                                        `tatttacactcacgaaaggaggataaccatatgtgttgggcagccgcaatacctatcgctatatctggCGCTATATCTGGCGCTCAGGCTATCAGTGGTCAGAACGCTCAGGCCAAAATGATTGCCGCTCAGACCGCTGCTGGTCGTCGTCAAGCTATGGAAATCATGCTGCAGACGAACATCCAGAATGCTGACCTATCGTTGCAAGCTCGAAGTCAACTTTAAGAAGCGTCCGCCGCGTTGACCCCACATAACATTCAGAAGGTCCAACCTATTTGGTCTATCCGCGCGGCTCtcggtg                                                                      >  1:293202‑M2/69‑299 (MQ=255)`  
                                                                                                                                                                                                                                         `atttacaatcacgaaaggaggataaccatatgtgttgggcagccgcaatacctatcgctatatctggCGCTATATCTGGCGCTCAGGCTATCAGTGGTCAGAACGCTCAGGCCAAAATGATTGCTGCTCAGACCGCTGCTGGTCGTCGTCAAGCTATGGAAATCATGAGGCAGACGAACATCCAGAATGCTGACCTATCGTTGCAAGCTCGAAGTAAACTTGAGGAAGCGTCCGCCGAGTTGACCCCACAGAACATGCAGAAGGTCCAAGCTATTGGGTCTATCCGAGCGGCTATCGgaga                                                                     <  1:56193‑M2/234‑1 (MQ=255)`  
                                                                                                                                                                                                                                                  `gtgtataggagacagaccatatgtgttggtcagccgcaatacctatcgctatatctggCACTATATCTGGCGCTCAGGCTATCAGTGGTCAGAACGCTCAGGCCAAAATGATTGCCGCTCAGACCGCTGCTGGTCGTCGTCAAGCTATGGAAATCAGGAGGCAGACGAACATCCAGAATGCTGACCGATCGTTGCAAGCTCGAAGTAAGCTTGAGGAAGCGTCCGCCGAGTTGACCCCACAGAACATGCAGAAGGTCCAAGCTATTGGGTCTATCCGAGCGGCTATCGGAGAGAGTATGCt                                                            <  1:55606‑M2/243‑1 (MQ=255)`  
                                                                                                                                                                                                                                                  `cacgaaaggaggataaccatatgtgttgggcagccgcaatacctatcgctatatctggCGCTATATCTGGCGCTCAGGCTATCAGTGGTCAGAACGCTCAGGCCAAAATGATTGCCGCTCAGACCGCTGCTGGTCGTCGTCAAGCTATGGAAATCATGAGGCAGACGAACATCCAGAATGCTGACCTATCGTTGCAAGCTCGAAGTAAACTTGAGTAAGCGTCCGACGAGTTGACCCCACAGAACATTCAGAAGGTCCAAGCTATTGGGTCTATCCGAGCggctatcggagagctgtcact                                                            >  1:304349‑M2/59‑293 (MQ=255)`  
                                                                                                                                                                                                                                                   `gtgtagaagagacagccattggtgttgggaagccgcaatacctatcgcgatatctggCTCTATATCTGGCGCTCAGGCTAGCAGTTGTCAGAACGCTCAGGCAAAAATGATTGCCGCTCAGACCGCTGCTGGTCGTCGTCAAGCTATGGAAATAATGAGGCAGACGAACATCCAGAATGCTGACCTATCGTTGCAAGCTCGAAGTAAACTTGAGGAAGCGTCCGCCGAGTTGACCTCACAGAACATGCAGAAGGTCCAAGCTATTGGGTCTATCCGAGCGGCTATCGGAGAGAGTATGCtt                                                           <  1:658572‑M2/244‑1 (MQ=255)`  
                                                                                                                                                                                                                                                        `aggaggataaccatatgtgttgggcagccgcaatacctatcgctatatctggCGCTATATCTGGCGCTCAGGCTATCAGTGGTCAGAACGCTCAGGCCAAAATGATTGCCGCTCAGACCGCTGCTGGTCGTCGTCAAGCTATGGAAATCATGAGGCAGACGAACATCCAGAATGCTGACCTATCGTTGCAAGCTCGAAGTAAACTTGAGGAAGCGTCCGCCGAGTTGACCCCACAGAACATGCAGAAGGTCCAAGCTATTGGGTCTATCCGAGCGGCTATCGGAGAGAGTATGCTTGAAgg                                                      <  1:573357‑M2/249‑1 (MQ=255)`  
                                                                                                                                                                                                                                                            `ggataaccatatgtgttgggcagccgcaatacctatcgctatatctggCGCTATATCTGGCGCTCAGGCTATCAGTGGTCAGAACGCTCAGGCCAAAATGATTGCCGCTCAGACCGCTGCTGGTCGTCGTCAAGCTATGGAAATCATGAGGCAGACGAACATCCAGAATGCTGACCTATCGTTGCAAGCTCGAAGTAAACTTGAGGAAGCGTCCGCCGAGTTGACCCCACAGAACATGCAGAAGGTCCAAGCTATTGGGTCTATCCGAGCGGCTATCGGAGAGAGTATGCTTGAAGATTcc                                                  <  1:5020‑M2/253‑1 (MQ=255)`  
                                                                                                                                                                                                                                                                 `agcatatgtgttgggcagccgcaatacctatcgctatatctggCGCTATATCTGGCGCTCAGGCTATCAGTGGTCAGAACGCTCAGGCCAAAATGATTGCCGCTCAGACCGCTGCTGGTCGTCGTCAAGCTATGGAAATCATGAGGCAGACGAACATCCAGAATGCTGACCTATCGTTGCAAGCTCGAAGTAAACTTGAGGAAGCGTCCGCCGAGTTGACCCCACAGAACATGCAGAAGGTCCAAGCTATTGGGTCTATCCGAGCGGCTATCGGAGAGAGTATGCTTGAAGGTTCCTCAAt                                             <  1:341532‑M2/258‑1 (MQ=255)`  
                                                                                                                                                                                                                                                                 `accatatgtgttgggcagccgcaatacctatcgctatatctggCGCTATATCTGGCTCTCAGGCTATCAGTGGTCAGAACGCTCAGGCCAAAATGCTTGCCGCTCAGACCGCTGCTGGTCGTCGTCAAGCTATGGAAATCATGAGGCAGCCGAACATCCAGCATGCTGACCTATCGTTGCAAGCTCGAAGTAAACTTGAGGAAGCGTCCGCCGAGTTGACCCCACAGAACATCCAGAAGGTCTAAGCTATTGGGTCTATCCTAGCGGCTCTAGGAGAGAGTatgcttgaaggctggctctt                                             >  1:581248‑M2/44‑292 (MQ=255)`  
                                                                                                                                                                                                                                                                     `tatgtgttgggcagccgcaatacctatcgctatatctggCGCTATATCTGGCGCTCAGGCTATCAGTGGTTATAACGCTCAGGCCAATTTGATTGCCGCTCAGACCGCTGCTGGTCGTCGTCAAGCTATGGAAATCATGAGGCAGACGAACTTTCAGAATGCTGACCTATTGTTTCAAGCTCGAAGTAAACTTGAGGACACGTCCGCCGAGTTGAACTCACAGAACCTGCAGAAGGTCCAAGCTATTGGGTCTATCCGAGCGGCTATCGGAGTGAGTATGCTTGAAGGTTCCTCAATGGAc                                         >  1:473004‑M2/40‑301 (MQ=255)`  
                                                                                                                                                                                                                                                                        `gtgttgggcagccgcaatacctatcgctatatctggCGCTATATCTGGCGCTCAGGCTATCAGTGGTCAGAACGCTCAGGCCAAAATGATTGCCGCTCAGACCGCTGCTGGTCGTCGTCAAGCTATGGAAATCATGAGGCAGACGAACATCCAGAATGCTGACCTATCGTTGCAAGCTCGAAGTAAACTTGAGGAAGCGTCCGCCGAGTTGACCCCACAGAACATGCAGAAGGTCCAAGCTATTGGGTCTATCCGAGAGGCTATCGGAGAGAGTATGCTTGAAGGTTCCTCAATGGACCGc                                      >  1:33635‑M2/37‑301 (MQ=255)`  
                                                                                                                                                                                                                                                                        `gtgttgggcagccgcaatacctatcgctatatctggCGCTATATCTGGCGATCAGGCTATCAGTGGTCAGAACGCTCAGGCCAAAATGATTGCCGCTCAGACCGCTGCTGGTCGTCGTCAAGCTATGGAAATCATGAGGCAGACGAACATCCAGAATGCTGACCTATCGTTGCAAGCTCGAAGTAAACTTGAGAAAGCGTCCGCAGCGTTGACCTCACAGAACATGAAGAAGGTCCAAGCTATTGGGTCTATCCGAGCGGCTATGGGGAAGAGTACTCTTTAaggttcctcactctgtctt                                      >  1:376857‑M2/37‑292 (MQ=255)`  
                                                                                                                                                                                                                                                                             `gggcagccgcaatacctatcgctatatctggCGCTATATCTGGCGCTCAGGCTATCAGTGGTCAGAACGCTCAGGCCAAAATGATTGCCGCTCAGACCGCTGCTGGTCGTCGTCAAGCTATGGAAATCATGAGGCAGACGAACATCCAGAATGCTGACCTATCGTTGCAAGCTCGAAGTAAACTTGAGGAAGCGTCCGCCGAGTTGACCCCACAGAACATGCAGAAGGTCCAAGCTATTGGGTCTATCCGAGCGGCTATCGGAGAGAGTATGCTTGAAGGTTCCTCAATGGACCGCATTaa                                 >  1:636696‑M2/32‑301 (MQ=255)`  
                                                                                                                                                                                                                                                                              `cgtgggctcggagatgtgtataagagaccgCGCTATATCTGGCGCTCAGGCTATCAGTGGTCAGAACGCTCAGGCCAAAATTATTGCCGCTCAGACCGCTGCTGGTCGTCGTCAAGCTATGGAAATCATGAGGCAGACGAACATCCAGAATGCTGACCTATCGTTGCAAGCTCGAAGTAAACTTGAGGAAGCGTCCGCCGAGTTGACCCCACAGAACATGCAGAAGGTCCAAGCTATTGGGTCTATCCGAGCGGCTATCGGAGAGAGTATGCTTGAAGGTTCCTCAATGGACCGCATTAAg                                <  1:174648‑M2/271‑1 (MQ=255)`  
                                                                                                                                                                                                                                                                               `gcagccgcaatacctatcgctatatctggCGCTATATCTGGCGCTCAGGCTATCAGTGGTCAGAACGCTCAGGCCAAAATGATTGCCGCTCAGACCGCTGCTGGTCGTCGTCAAGCTATGGAAATCATGAGGCAGACGAACATCCAGAATGCTGACCTATCGTTGCAAGCTCGAAGTAAACTTGAGGAAGCGTCCGCCGAGTTGACCCCACAGAACATGCAGAAGGTCCAAGCTATTGGGTCTATCCGAGCGGCTATCGGAGAGAGTATGCTTGAAGGTTCCTCAATGGACCGCATTAAGc                               <  1:543189‑M2/272‑1 (MQ=255)`  
                                                                                                                                                                                                                                                                                    `cgcaatacctatcgatatatctggCGCTATAGCTGGCGCTCAGGCTATCAGTGGTCAGAACGCTCAGGCCAAAATGATTGCCGCTCAGACCGCTGCTGGTCGTCGTCAAGCTATGGAAATCATGAGGCAGACGAACATCCAGAATGCTGACCTATCGTTGCAAGCTCGAAGTAAACTTGAGGAAGCGTCCGCCGAGTTGACCCCACAGAACATGCAGAAGGTCCAAGCTATTGGGTCTATCCGAGCGGCTATCGGAGAGAGTATGCTTGAAGGTTCCTCAATGGACCGCATTAAGCGAGTc                          <  1:249664‑M2/277‑1 (MQ=255)`  
                                                                                                                                                                                                                                                                                     `gcaatacctatcgctatatctggCGCTATATCTGGCGCTCAGGCTATCAGTGGTCAGAACGCTCAGGCCAAAATGATTGCCGCTCAGACCGCTGCTGGTCGTCGTCAAGCTATGGAAATCATGAGGCAGACGAACATCCAGAATGCTGACCTATCGTTGCAAGCTCGAAGTAAACTTGAGGAAGCGTCCGCCGAGTTGACCCCACAGAACATGCAGAAGTTCCAAGCTCTTGGGTCTATCCGAGCGGCTATCGGAGAGATTATGCTTGAAGGTTCCTCAATGGACCGCATTTAGCGAGtcc                         >  1:412920‑M2/24‑300 (MQ=255)`  
                                                                                                                                                                                                                                                                                         `aacctatcgctatagctggCGCTATATCTGGCGCTCAGGCTATCAGTTGTCAGAACGATAAGGCCAAAATGATTGAAGCTCAGACCGGTGCTGGTCGTCGTAAAGCTATGGAAATCATGAGGCAGACGAACATCCAGAATGCTGACCTATCGTTGCAAGCTCGAAGTAAACTTGAGGAAGCGTCCGCCGAGTTGAACCCACAGAACATGCAGAAGGTCCAAGCTATTGGGTCTATCCGAGCGGCTATCGGAGAGAGTATGCTTGAAGGTTCCTCAATGGACCGCATTAAGCGAGTCACAGa                     <  1:100163‑M2/282‑1 (MQ=255)`  
                                                                                                                                                                                                                                                                                          `acctatcgctatatctggCGCTATATCTGGCGCTCAGTCTATCAGTGGTCAGAACGCTCAGGCCAAAATGATTGCCGCTCAGACCGCTGCTGGTCGTCGTCAAGCTATGGAAATCATGAGGCAGACGAACATCCAGAGTGCTGACCTATCGTTGCAAGCTCGAAGTAAACTTGAGGAAGCGTCCGCCGAGTTGACCCCACAGAACATGCAAAAGGTCCAAGCTATTGGGTCTATCCGAGCGGCTATCTGAGAGAGTATGCTTGAAGGTTCCTCAATGGACCGCATTAAGCGAGTCACAGaa                    >  1:280474‑M2/19‑301 (MQ=255)`  
                                                                                                                                                                                                                                                                                              `caggatatatctggCGCTATATCTGGCGCTCAGGCTATCAGTGGTCAGAACGCTCAGGCCAAAATGATTGCCGCTCAGACCGCTGCTGGTCGTCGTCAAGCTATGGAAATCATGAGGCAGACGAACATCCAGAATGCTGACCTATCGTTGCAAGCTCGAAGTAAACTTGAGGAAGCGTCCGCCGAGTTGACCCCACAGAACATGCAGAAGGTCCAAGCTATTGGGTCTATCCGAGCGGCTATCGGAGAGAGTATGCTTGAAGGTTCCTCAATGGACCGCATTAAGCGAGTCACAGAAGGAc                <  1:264133‑M2/287‑1 (MQ=255)`  
                                                                                                                                                                                                                                                                                              `atcgctatatctggCGCAATATCTGGGGCTCAGGCTATCAGGGGTCAGAACGCTAAGGCCAAAATGATTGCAGCTAAGACCGCTGCTGGTCGTCGTCAAGCTATGGAAATCATGAGGCAGACGAACATCCAGAATGCTGACCTATCGTTGCAAGCTCGAAGTAAACTTGAGGAAGCGTCCGCCGAGTTGACCCCACAGAACATGCAGAAGGTCCAAGCTATTGGGTCTATCCGAGCGGCTATCGGAGAGAGTATGCTTGAAGGTTCCTCAATGGACCGCATTAAGCGAGTCACAGAAGGAc                <  1:433858‑M2/287‑1 (MQ=255)`  
                                                                                                                                                                                                                                                                                               `tcgctatatctggCGCTATATCTGGCGCTCAGGCTATCAGTGGTCAGAACGCTCAGGCCAAAATGATTGCCGCTCAGACCGCTGCTGGTCGTCGTCAAGCTATGGAAATCATGAGGCAGACGAACATCCAGAATGCTGACCTATCGTTGCAAGCTCGAAGTAAACTTGAGGAAGCGTCCGCCGAGTTGACCCCACAGAACATGCAGAAGGTCCAAGCTATTGGGTCTATCCGAGCGGCTATCGGAGATAGTATCCTTGAAGGTTCCTCAAtggaccgcattaagcgctgcctcttatacac               >  1:630259‑M2/14‑286 (MQ=255)`  
                                                                                                                                                                                                                                                                                               `tcgctatatctggCGCTATATCTGGCGCTCAGGCTATCAGTGGTCAGAACGCTCAGGCCAAAATGATTGCCGCTCAGACCGCTGCTGGTCGTCGTCAAGCTATGGAAATCATGAGGCAGACGAACATCCAGAATGCTGACCTATCGTTGCAAGCTCGAAGTAAACTTGAGGAAGCGTCCGCCGAGTTGACCCCACAGAACATGCAGAAGGTACAAGCTATTGGGTCTATCCGAGAGGCCATCGGAGAGAGTATGCTTGCAGGTTCCTCAATGGACCGAATTAAGCGAGTCACAGAAGGCCa               >  1:155257‑M2/14‑301 (MQ=255)`  
                                                                                                                                                                                                                                                                                                `cgctatatctggCGCTATATCTGGCGCTCAGGCTATCAGTGGTCAGAACGCTCAGGCCAAAATGATTGCCACTCAGACCGCTGCTGGTCGTCGTCAAGCTATGGAAATCATGAGGCAGACGAACATCCAGAATGCTGACCTCTCGTTGCAAGCTCGAAGTAAACTTGAGGAAGCGTCCGCCGAGTTGACCCCACAGAACATGCAGAAGGTCCAAGCTATTGGGTCTATCCGAGCGGCTATCGGAGAGAGTATGCTTGAAGGTTCCTACATGGACCGCATTAAGCGAGTCAAAGAAGgaccg              >  1:642643‑M2/13‑299 (MQ=255)`  
                                                                                                                                                                                                                                                                                                 `gctatatctggCGCTATATCTGGCGCTCAGGCTATCAGTGGTCAGAACGCTCAGGCCAAAATGATTGCCGCTCAGACCGCTGCTGGTCGTCGTCAAGCTATGGAAATCATGAGGCAGACGAACATCCAGAATGCTGCCCTATCGTTGCAAGCTCGAAGTAAACTTGAGGAAGCGTACGCCGAGTTGACCCCACAGAACATGCAGAAGGTCCAAGCTATTGGGTATATCCGAGCGGCTATCGGAGAGAGTATGCTTGAAGGTTCCTCAATGGACCGCATTAAGCGAATCACAGAAGGACAGt             >  1:479358‑M2/12‑301 (MQ=255)`  
                                                                                                                                                                                                                                                                                                     `tatctggCGCGATATCTGGCGCGCAGGCTATCAGTGGACAGAACGCTCAGGCCAAAATGATTGCCGCTCAGACCGATGCTGGTCGTCGTAAAGCTATGGAAATCATGAGGCAGACGAACATCCAGAATGCTGACATATCGTTGCAAGCTCGAAGTAAACTTGAGGAAGCGTCCGCCGAGTTGACCCCACAGAACATGCAGAAGGTCCAAGCTATTGGGTCTATCCGAGCGGCTATCGGACAGAGTATGCTTGAAGGTTCCTCAATGGACCGCATTAAGCGAGTCACAGAAGGACAGTTCAt         <  1:186953‑M2/294‑1 (MQ=255)`  
                                                                                                                                                                                                                                                                                                      `atctggCGCTATATCTGGCGCTCAGGCTATCAGTGGTCAGAACGCTCAGGCCAAAATGATTGCCGCTCAGACCGCTGCTGGTCGTCGTCAAGCTATGGAAATCATGAGGCAGACGAACATCCAGAATGCTGACCTATCGTTGCAAGCTCGAAGTAAACTTGAGGAAGCGTCCGCCGAGTTGACCCCACAGAACATGCAGAAGGTCCAAGCTATTGGGTCTATCCGAGCGGCTATCGGAGAGAGTATGCTTGAAGGTTCCTCAATGGACCGCATTAAGCGAGTCACAGAAGGACAGTTCAtt        >  1:244664‑M2/7‑301 (MQ=255)`  
                                                                                                                                                                                                                                                                                                      `atctggCGCTATATCTGGCGCTCAGGCTATCAGTGGTCAGAACGCTCAGGCCAAAATGATTGCCGCTCAGACCGCTGCTGGTCGTCGTCAAGCTATGGAAATCATGAGGCAGACGAACATCCAGAATGCTGACCTATCGTTGCAAGATCGAAGTAAACTTGAGGAAGCGTACGCCGAGTTGACCCCACAGAACATGCAGAAGGTCCAAGCTATTGGGTCTATCCGAGCGGCTATCGGAGAGAGTATGCTTGAAGGTTCCTCAATGGACCGCATTAAGCGATTCACAGAAGGACAGATCAtt        >  1:550188‑M2/7‑301 (MQ=255)`  
                                                                                                                                                                                                                                                                                                      `agctggCGCTATATCTGGCGCTCAGGCTATCAGTGGTCAGAACGCTCAGGCCAAAATGATTGCCGCTCAGACCGCTGCTGGTCGTCGTCAAGCTATGGAAATCATGAGGCAGACGAACATCCAGAATGCTGACCTATCGTTGCAAGCTCGAAGTAAACTTGAGGAAGCGTCCGCCGAGTTGACCCCACAGAACATGCAGAAGGTCCAAGCTATTGGGTCTATCCGAGCGGCTATCGGAGAGAGTATGCTTGAAGGTTCCTCAATGGACCGCATTAAGCGAGTCACAGAAGGACAGTTCAtt        <  1:21519‑M2/295‑1 (MQ=255)`  
                                                                                                                                                                                                                                                                                                          `ggCGCTATATCTGGCGCTCAGGCTATCAGTGGTCAGAACGCTCAGGCCAAAATGATTGCCGCTCAGACCGCTGCTGGTCGTCGTCAAGCTATGGAAATCATGAGGCAGACGAACATCCAGAATGCTGACCTATCGTTGCAAGCTCGAAGTAAACTTGAGGAAGCGTCCGCCGAGTTGACCCCACAGAACATGCAGAAGGTCCAAGCTATTGGGTCTATCCGAGCGGCTATCGGAACGAGTATGCTTGAAGGTTCCTCAATGGACCGCATTAAGCGAGTCACAGAAGGACCGTTCATTCggg    >  1:461953‑M2/3‑301 (MQ=255)`  

                                                                                                                                                                                                                                                                                                            `|`                                                                                                                                                                                                                                                                                                              
`CTAGCTATCGGTGGTAACTGCGGGGACCAGTGCTGGTTCGTTACGAGCGACCAAGTGTGGCGACTTAGTGGAAAGGCTAAGCGAAAGTTCCGTAAGTTAATCATGGAGTATCGCGATAAGATGCTTGAGAAGTATGATACTCTTTGGAATTACGTATGGGTAGGCAATACGTCCCACATTCGTTTCCTCAAGACTATCGGTGCGGTATTCCATGAAGAGTACACACGAGATGGTCAATTTCAGTTATTTACAATCACGAAAGGAGGATAACCATATGTGTTGGGCAGCCGCAATACCTATCGCTATATCTGGCGCTCAGGCTATCAGTGGTCAGAACGCTCAGGCCAAAATGATTGCCGCTCAGACCGCTGCTGGTCGTCGTCAAGCTATGGAAATCATGAGGCAGACGAACATCCAGAATGCTGACCTATCGTTGCAAGCTCGAAGTAAACTTGAGGAAGCGTCCGCCGAGTTGACCTCACAGAACATGCAGAAGGTCCAAGCTATTGGGTCTATCCGAGCGGCTATCGGAGAGAGTATGCTTGAAGGTTCCTCAATGGACCGCATTAAGCGAGTCACAGAAGGACAGTTCATTCGGGAA  >  NC_001604.1/27454‑28054`

  

Alignment Legend

Aligned base mismatch/match (shaded by quality score): `ATCG/ATCG` `< 3 ≤` `ATCG/ATCG` `< 9 ≤` `ATCG/ATCG` `< 19 ≤` `ATCG/ATCG` `< 33 ≤` `ATCG/ATCG` `< 38 ≤` `ATCG/ATCG`

Unaligned base: `atcg`    Masked matching base: `atcg`    Alignment gap: `‑`    Deleted base: `‑`

Reads not counted as support for junction

read\_name Not counted due to insufficient overlap past the breakpoint.

read\_name Not counted due to not crossing MOB target site duplication.
